# A germline mutation in *SRRM2*, a splicing factor gene, is implicated in papillary thyroid carcinoma predisposition

**DOI:** 10.1038/srep10566

**Published:** 2015-07-02

**Authors:** Jerneja Tomsic, Huiling He, Keiko Akagi, Sandya Liyanarachchi, Qun Pan, Blake Bertani, Rebecca Nagy, David E. Symer, Benjamin J. Blencowe, Albert de la Chapelle

**Affiliations:** 1 Department of Molecular Virology, Immunology and Medical Genetics, Ohio State University Wexner Medical Center and Comprehensive Cancer Center, Ohio State University, Columbus, Ohio, United States of America; 2 Banting and Best Department of Medical Research, University of Toronto, Toronto, Canada; 3 Department of Internal Medicine, Ohio State University Wexner Medical Center and Comprehensive Cancer Center, Ohio State University, Columbus, Ohio, United States of America; 4 Department of Biomedical Informatics, Ohio State University Wexner Medical Center and Comprehensive Cancer Center, the Ohio State University, Columbus, Ohio, United States of America; 5 Department of Molecular Genetics, University of Toronto, Toronto, Canada

## Abstract

Papillary thyroid carcinoma (PTC) displays strong but so far largely uncharacterized heritability. Here we studied genetic predisposition in a family with six affected individuals. We genotyped all available family members and conducted whole exome sequencing of blood DNA from two affected individuals. Haplotype analysis and other genetic criteria narrowed our list of candidates to a germline variant in the serine/arginine repetitive matrix 2 gene (*SRRM2*). This heterozygous variant, c.1037C > T (Ser346Phe or S346F; rs149019598) cosegregated with PTC in the family. It was not found in 138 other PTC families. It was found in 7/1,170 sporadic PTC cases and in 0/1,404 controls (p = 0.004). The encoded protein SRRM2 (also called SRm300) is part of the RNA splicing machinery. To evaluate the possibility that the S346F missense mutation affects alternative splicing, we compared RNA-Seq data in leukocytes from three mutation carriers and three controls. Significant differences in alternative splicing were identified for 1,642 exons, of which a subset of 7 exons was verified experimentally. The results confirmed a higher ratio of inclusion of exons in mutation carriers. These data suggest that the S346F mutation in SRRM2 predisposes to PTC by affecting alternative splicing of unidentified downstream target genes.

Thyroid cancer is the ninth most common cancer in the United States, and its incidence is rising (National Cancer Institute; http://seer.cancer.gov/statfacts/html/thyro.html)^1^,^2^. There are four distinct types of thyroid cancer, namely papillary (PTC, MIM 188550), follicular (FTC, MIM 188470), anaplastic (ATC) and medullary thyroid cancers (MTC, MIM 155240). PTC, FTC and ATC are known as non-medullary thyroid cancers (NMTC), and account for >90% of all cases. PTC accounts for a large majority of NMTC and has an excellent prognosis (10-year survival >90%), but morbidity from this type is high, mainly due to local spread and complications of therapeutic measures.

A relatively high number of PTC cases (i.e. 3–10%) are familial^3^, although most are sporadic. Based on case-control studies, the familiality of PTC ranks among the highest of all cancer types. In early work reported from Utah and Sweden, the risk in first-degree relatives was as high as 8-12 fold^4^,^5^,^6^. More recently, similar or lower risk estimates have been reported, nevertheless confirming that PTC is one of the most heritable of all cancers^7^,^8^,^9^,^10^.

Historically, studies seeking the genetic factors predisposing to PTC have involved linkage analysis of affected families^11^,^12^,^13^,^14^,^15^. While causative mutations were not identified in early reports^16^, more recent linkage studies have reported the discovery of mutations in several culpable or candidate genes. These include an ultra-rare mutation in an enhancer element on chromosome 4, found to predispose to PTC in a large family^17^. A mutation in a non-coding RNA gene embedded in *TG* (chromosome 8) also is likely to predispose to PTC^18^. Mutations in *SRGAP1* (chromosome 12) contribute to genetic predisposition with a relatively high penetrance^19^, but they occur in only small proportions of familial and sporadic cases. A germline missense mutation in *NKX2.1* (also known as thyroid transcription factor 1) on chromosome 14 was found in four out of 20 PTC patients with a history of multinodular goiter, but this change was not found in PTC patients without multinodular goiter^20^. A common SNP in the microRNA-146a gene was shown to predispose to PTC with low penetrance^21^,^22^. Taken together, these results suggest that particular mutations contributing to PTC at high- or medium-high penetrance may be inherited following a Mendelian pattern, although such cases occur rarely. More recently, this notion has been reinforced, at least in part, by results of genome-wide association studies (GWAS), whereby several additional low-penetrance risk variants have been identified. One SNP detected by GWAS turned out to inhibit profoundly the expression of a thyroid-specific long intergenic noncoding RNA (lincRNA) located close to *NKX2.1*^23^. It has been estimated that the five best-characterized SNPs identified in GWAS may contribute to 11% of PTC familiality^24^.

Clearly, many more germline mutations predisposing to heritable PTC remain to be found. In this study, we have searched for previously unidentified variants by combining genotyping, haplotype analysis, whole exome sequencing (WES) and genetic linkage analysis in a well-documented PTC family. Such combined analysis led us to identify a variant c.1037C > T (Ser346Phe) in the splicing gene *SRRM2*, making it a leading candidate mutation contributing to PTC in this family. Taken together with earlier efforts by our group and others, these results further emphasize the genetic heterogeneity of PTC predisposition. The results are compatible with a recurring theme in studies of genetic predisposition, called “clan genomics”. This view holds that rare variants in populations together may account for much of the genetic risk for common genetic diseases^25^.

## Results

### Identification of genetic variants in a PTC family

**Figure 1:**
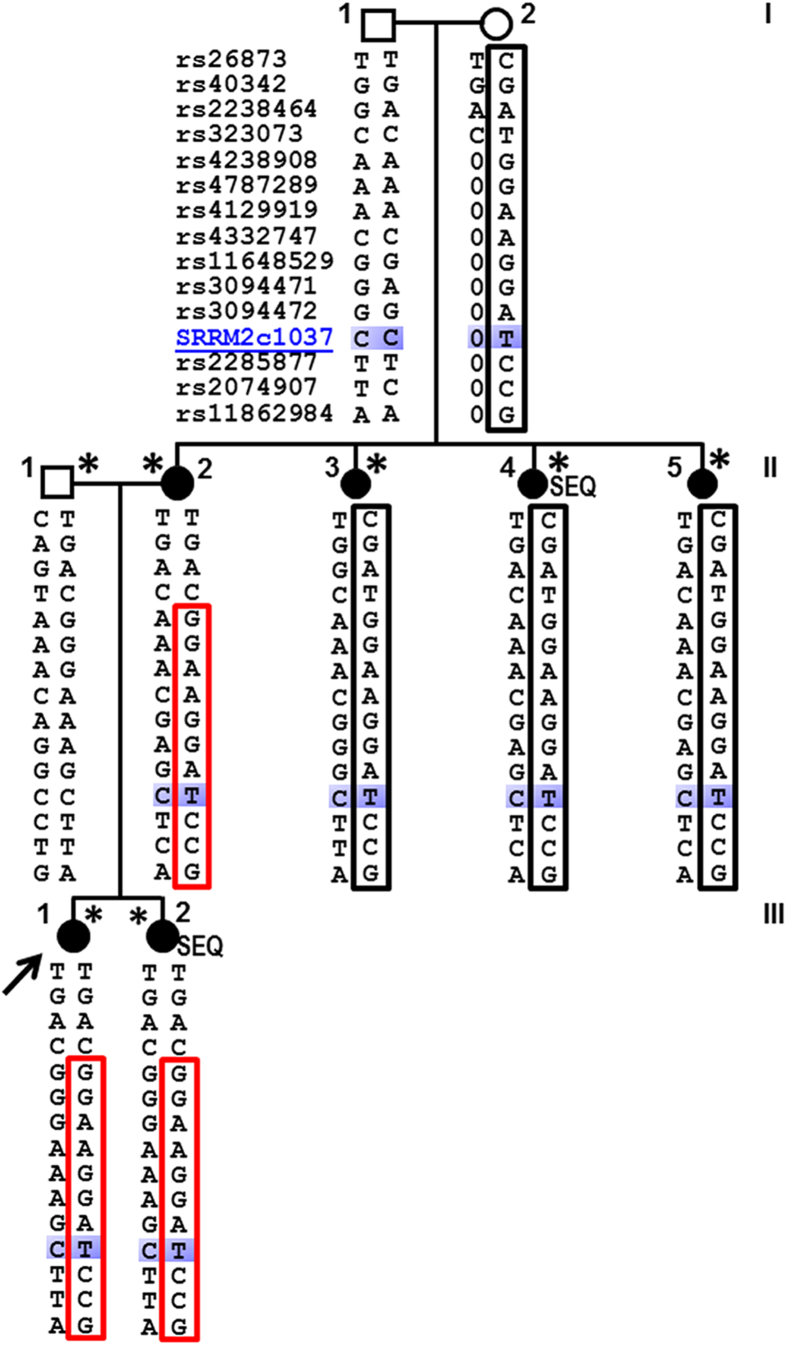
Pedigree, haplotypes and the missense mutation. A unique haplotype at *SRRM2* (*red box*) cosegregates with the PTC phenotype in the family. *Solid circles*, individuals affected with PTC; *arrow*, proband; *SEQ,* individuals studied by whole exome sequencing; *, individuals studied for linkage. Samples from individuals I,1 and I,2 were not available for genotyping. SRRM2c1037 indicates the mutation studied (also known as rs149019598). The shorter haplotype (*red box*) measures 491 kb.

Upon realizing that mutations predisposing to PTC or NMTC may be rare or even unique to a single family^25^, we chose to study a large PTC family with six affected first- or second-degree relatives in whom the pattern of inheritance was Mendelian or Mendelian–like (Fig. 1). In this family, three of the affected individuals were ≤25 years old (Supplementary Table 1).

All members of this PTC family were genotyped using a commercial single nucleotide polymorphism (SNP) array, which included approximately 220,000 SNPs with the highest tagging power (cf. Materials and Methods). This was done despite our previous experience in families with similar genetic architectures^18^ suggesting that this approach would produce numerous low genetic linkage peaks that for the most part are irrelevant “noise”.

At the same time, we used WES to identify potential variants affecting protein-coding genes, by analyzing blood (germline) DNA samples from two affected individuals of the family. After aligning the raw sequence data and applying standard quality assurance procedures, we identified candidate variants using bioinformatics software including Samtools and BCFtools (cf. Materials and Methods).

### Ranking candidate variants using linkage and haplotype analysis

Each predicted variant that was identified from WES data was filtered further using several criteria, including its shared presence in both family members with PTC and its population allele frequency of <2% as observed in 1000 Genomes project phase 1 data. We assessed the potential functional consequences of candidate variants using the Ensembl variant effect predictor. We also required that variant genes that would be chosen for further study had to be expressed in thyroid tissue (Ref. 26 and http://cgap.nci.nih.gov/SAGE/AnatomicViewer). At this point, only 21 likely candidate variants (in 21 genes) remained for further consideration (Table 1).

Using the SNP genotyping data from all family members, we performed genetic linkage analysis. This resulted in multiple, low and wide linkage peaks, covering major parts of numerous chromosomes (Supplementary Fig. 1). Also as expected, no or low linkage peaks were found in many other chromosomal regions. When candidate variants mapped to these latter regions of low or absent linkage, we ranked them lower on our list of potential predisposing factors.

Combining our SNP genotyping dataset with WES variant data, we constructed haplotypes flanking the 21 candidate variants (Table 1). This haplotype analysis resulted in the exclusion of all but two of them, i.e. in *SRRM2* and *CHD9.* The same haplotypes were observed in all affected family members at these two remaining candidate loci.

### A recurrent mutation in SRRM2

As another step in refining our list of top candidate variants, we used Sanger sequencing to confirm the presence of the candidate exonic variants in the two genes, *SRRM2* and *CHD9* (Supplementary Fig. 2a). This analysis also confirmed the segregation of the variants with PTC in all available samples from the family. The *SRRM2* variant was further confirmed by sequencing RNA extracted from blood (Supplementary Fig. 2b).

We hypothesized that a rare, high-penetrance mutation could predispose to PTC in this family^27^. To evaluate the same two remaining variant candidates in broader populations, we determined their frequencies both in sporadic PTC cases and in unaffected controls. Our initial results pinpointed the candidate gene *SRRM2* as the most promising, as its variant was present in two out of 187 sporadic PTC cases but in zero out of 189 controls. When we added additional cases and controls, the *SRRM2* mutation was found in a total of seven out of 1170 sporadic PTC cases, but in zero of 1404 controls (p = 0.004). It was also not found in 138 familial cases. By contrast, the variant in *CHD9* was completely absent from all further samples studied; that is, it was present in zero of 720 sporadic PTC cases and in zero of 727 controls. These results do not exclude *CHD9* gene as a plausible candidate, as it could still be an ultra-rare predisposing genetic variant that was detected in just the one PTC family under study. In summary, the variant in the *SRRM2* coding sequence segregates with the PTC phenotype in all affected individuals in the family; it is flanked by a large haplotype encompassing the mutation; and it is significantly enriched in sporadic PTC cases.

The seven sporadic cases harboring the same mutation in *SRRM2* each displayed classic PTC histology (Supplementary Table 1). We reviewed the self-reported family histories for cancer and/or benign thyroid disease for all first- or second-degree relatives of these seven individuals, wherever available. However, none of these seven PTC patients reported any family history of PTC (Supplementary Table 1). Moreover, their rate of benign thyroid disease was no greater than what would be expected in the general population.

### Missense variant S346F affects SRRM2 function

**Figure 2:**
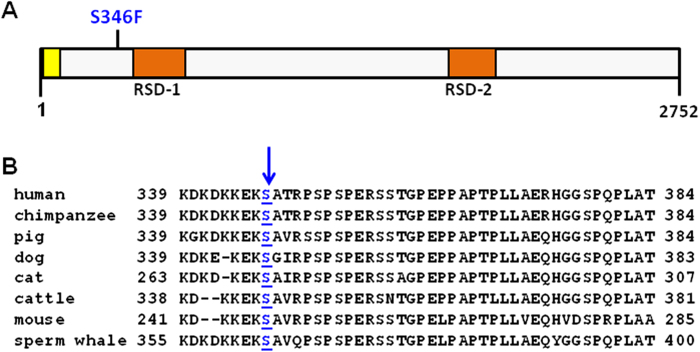
SRRM2 protein. (**a**) Schematic drawing of human SRRM2 protein indicating the position of the candidate mutation Ser346Phe (S346F). Protein domains were based on information in. Yellow, RNA binding domain (residues 92 - 184); *orange*, arginine-serine rich domains RSD-1 and RSD-2 (residues 481 - 719 and 1838 – 2058 respectively). (**b**) SRRM2 protein sequence conservation among eight species of mammals: human (NP_057417.3), chimpanzee (XP_003314977.1), pig (NP_001231474.1), dog (XP_005621725.1), cat (XP_006942505.1), cattle (XP_002697976.2), mouse (NP_780438.2), sperm whale (XP_007100096.1). *Blue arrow*, highly conserved Ser346 underlined.

The missense mutation c.1037C > T in exon 11 in *SRRM2* is predicted to change the encoded amino acid from serine to phenylalanine (S346F; Fig. 2a).This variant is a previously reported single nucleotide polymorphism (SNP), rs149019598. It was detected at 0.09% frequency in the European-American population by the Exome Sequencing Project (ESP; https://esp.gs.washington.edu/drupal/). This missense mutation is highly conserved among different species of mammals (Fig. 2b). It has been predicted by SIFT^28^ to be damaging and by PolyPhen-2^29^ to be probably damaging.

SRRM2 (also known as SRm300) is a splicing factor with a molecular weight of ~300 kDa^30^. It was shown to associate with SRm160, forming the SRm160/SRm300 splicing coactivator^31^, a core component of the spliceosome^32^. SRRM2 promotes exon enhancer-dependent splicing by forming multiple critical interactions with factors bound directly to pre-mRNA, including SR family proteins and U2 snRNP^33^. Interestingly, the yeast ortholog of SRRM2, Cwc21, also associates with U2 snRNP and assembled spliceosomes, and promotes splicing^34^,^35^. Although the precise role of SRm300 in splicing is not known, it has been proposed to function through its interaction with critical splicing factors in the control of alternative splicing (AS; 30).

We therefore hypothesized that the S346F mutation in SRRM2 could result in changes in the alternative splicing of a limited number of transcripts, e.g. those that are SRRM2-dependent or perhaps those specifically interacting with S346 and surrounding amino acids. To test this hypothesis, we performed high-throughput RNA sequencing (RNA-Seq) of polyadenylated RNA transcripts extracted from blood samples obtained from three PTC patients heterozygous for the *SRRM2* mutation and from three wildtype controls. In analyzing the RNA-Seq data, we defined each internal exon in annotated, spliced transcripts as the potential “cassette” or alternative exon. Thus each alternative splicing event involving such a cassette was defined by three exons: C1, A and C2 (Supplementary Fig. 3), where A is the alternative exon, flanked by the upstream exon C1 as the 5´ constitutive exon and downstream exon C2 as the 3´ constitutive exon. Accordingly, we compared the expression of the two constitutive or “included” junctions, relative to the one alternative (or “skipped”) junction. We created a database of the resulting, non-redundant splice junction sequences (i.e., C1A, AC2 and C1C2; Supplementary Fig. 3), formed by alternatively including or skipping each exon A. Thus this database enumerated both of the resulting, alternatively spliced isoforms. RNA-Seq reads were compared to this junction database to determine “percent spliced in” (PSI) values (Materials and Methods^36^). RT-PCR was used to confirm the data obtained by RNA-Seq. In this assay PSI values were defined as the ratio of the “included” expression level vs. the sum of both spliced isoforms.

**Figure 3:**
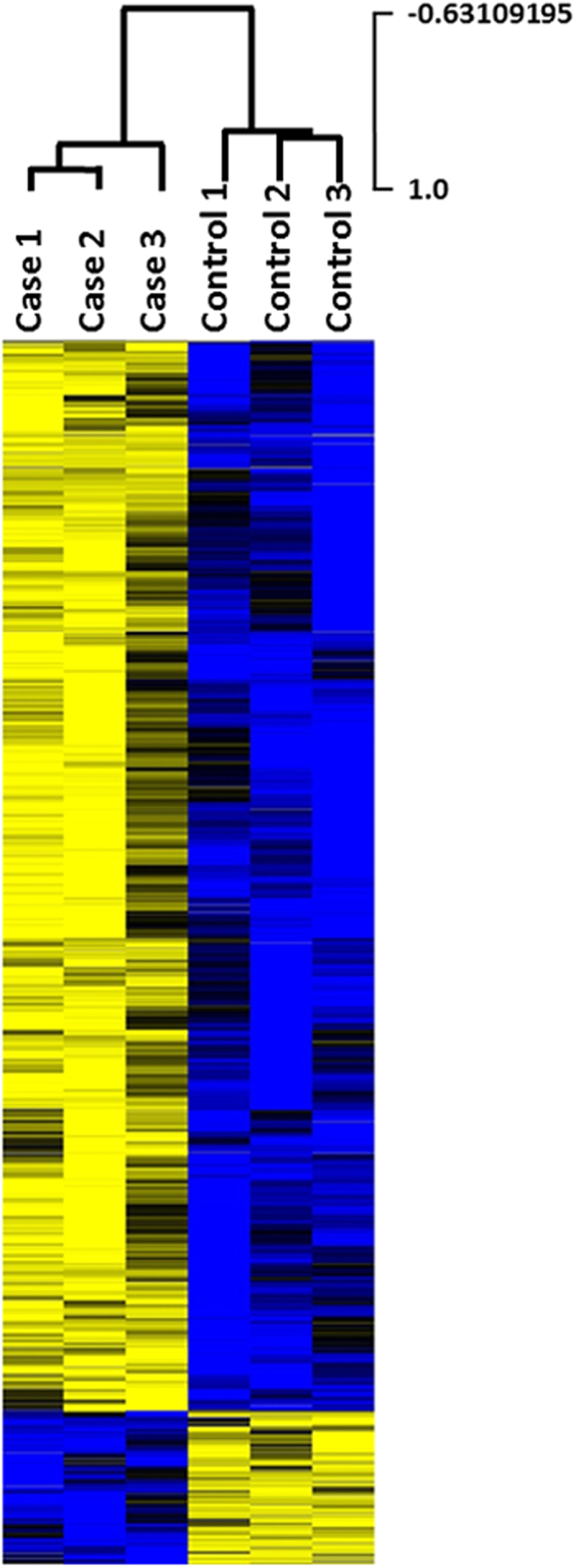
Heat map of 1642 alternative splicing events. Hierarchical cluster analysis was performed using MeV software (http://www.tm4.org), with normalized, median-centered PSI values. Pearson correlation similarity metric and average linkage clustering methods were used. Most events present higher PSI in cases vs controls. *Yellow*, higher PSI; *blue*, lower PSI.

To determine the potential impact of the S346F variant in affecting AS, we then calculated a t-test statistic to compare the average PSI values in cases vs controls. Those alternatively spliced transcripts with a p-value cutoff of 0.05 were retained for additional downstream analysis. There were 1,642 splicing events in which alternatively spliced transcripts in the cases were differentially expressed when compared to controls (Fig. 3).

We then further validated some of these events predicted from the RNA-Seq data by selecting an arbitrary set of seven differentially spliced genes (Table 2). In these cases, we required a difference in the average PSI values between cases and controls of at least 20% based on RNA-Seq. The differential expression of the two isoforms was assayed using semi-quantitative RT-PCR. Constrained by the limited number of available samples harboring a variant in *SRRM2*, we were able to test five cases, whereas we tested nine controls. Two of the cases and one of the controls were also studied in the initial RNA-Seq experiments.

**Figure 4:**
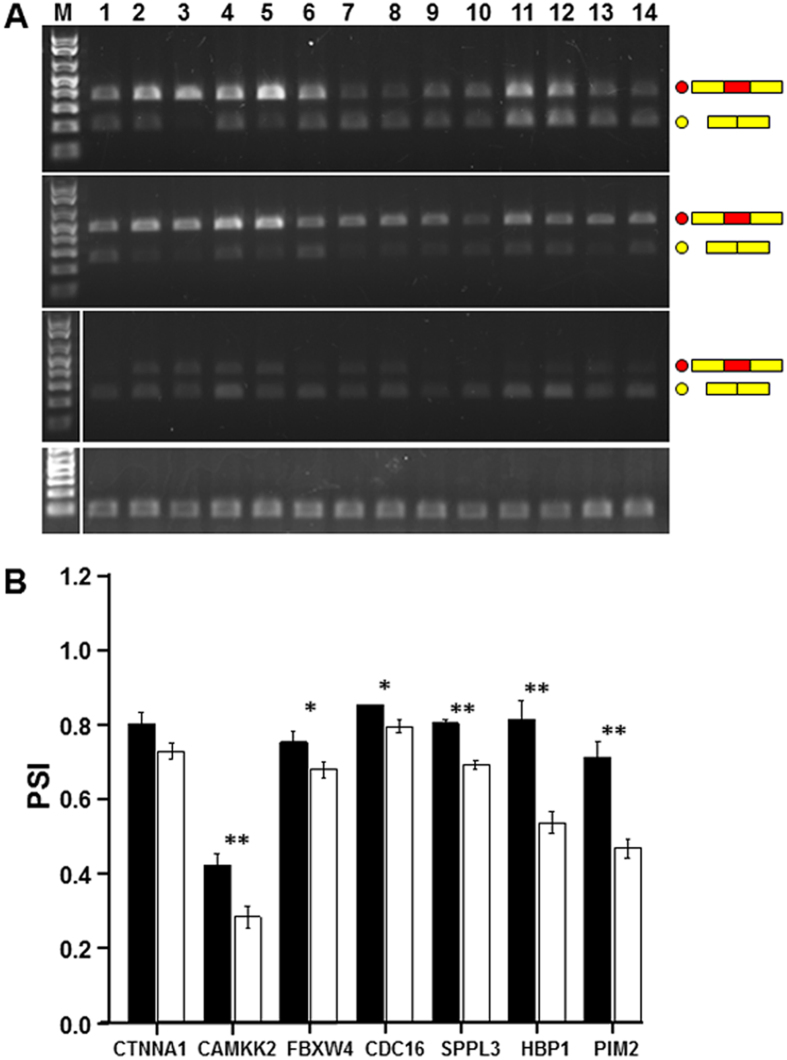
Analysis of alternative splicing (AS) in cases (carrying the SRRM2 S346F mutation) and controls. (**a**) Semi-quantitative RT-PCR assays were performed using blood RNA from 5 cases heterozygous for the SRRM2 S346F mutation (lanes 1-5) and 9 controls (lanes 6-14). A representative agarose gel shows AS in *HBP1* (first panel), *CTNNA1* (second panel), *CAMKK2* (third panel), and GAPDH (fourth panel). *M*, molecular weight marker (1 kb Plus, Invitrogen); *red and yellow dots*: exon-included and exon-skipped isoforms, respectively. (**b**) Bar plots represent “percent spliced in” (PSI) values obtained by end-point RT-PCR for seven arbitrarily selected genes. Differences in PSI were significant in six out of seven genes studied. Error bars represent ± SEM. *Black bars*, cases (n = 5); *white bars*, controls (n = 9). *, 0.05 > p-value > 0.005; **, p-value < 0.005.

Primers were designed to anneal to the two exons flanking the predicted, differentially included exon (Supplementary Table 2). Complementary DNA (cDNA) was synthesized, end-point RT-PCR was performed, and PCR products were visualized on a gel (Fig. 4a). The two alternatively spliced bands were quantified.

Average PSI values in cases and controls, as evaluated by RT-PCR for seven genes, were plotted in Fig. 4b and reported in Supplementary Table 3. For six of these seven genes, the PSI values were significantly different in comparing the cases vs. controls. Meanwhile, the PSI difference for the remaining gene, i.e. *CTNNA1*, was borderline significant (Fig. 4b).

To compare PSI values measured by RNA-Seq vs. values measured by RT-PCR, we performed a Pearson correlation and Spearman rank correlation analysis for all seven genes in the cases and controls combined. Statistically significant correlations were obtained in both cases (Pearson correlation: cor = 0.865, p-value = 6.373e-05; Spearman rank correlation: cor = 0.798, p-value = 0.001). A resulting scatterplot, showing an association between these methods, is presented in Supplementary Fig. 4.

## Discussion

The high heritability of PTC is likely due to the combined contributions of rare but high-penetrance mutations in some cases, and common but low-penetrance variants in others. The latter are best searched for by GWAS, which was not used here. By contrast, the higher-penetrance mutations are likely to underlie disease clustering in families, displaying a pattern of Mendelian inheritance. Using a combination of genetic methods, we have detected and validated a mutation in a single candidate gene, *SRRM2*, which appears very likely to explain PTC in the one family studied (Fig. 1). This result is similar to findings by numerous researchers applying these methods in familial diseases^27^,^37^,^38^,^39^,^40^,^41^. We are currently using similar combinations of genetic methods to identify the likely candidate genes in additional PTC families.

Traditionally, such mutations have been mapped by linkage analysis. Unfortunately, this approach alone in PTC has been remarkably unsuccessful^16^,^42^. The simplest explanation is that the number of affected individuals is usually too small to be informative, given the presumptive genetic heterogeneity of PTC. Thus different families would be expected to harbor rare, distinctive genetic variants, consistent with the principle of “clan genomics”^25^.

We used WES as a primary screen to detect candidate variants which segregated with the PTC phenotype in a family. We chose this single family with 6 closely related, affected individuals because of the Mendelian-like segregation of the disease. We postulated that integrating WES together with linkage data would allow us to rank the medium- to high-penetrance candidate variants affecting genes. Subsequently, after their initial identification, such protein-coding genetic variants could be filtered by other criteria and scrutinized experimentally to prioritize them further, according to their potential contributions to PTC pathogenesis.

An analogous WES approach already has led to the classification of the genetic basis of numerous diseases such as metachondromatosis^43^ and autism^44^. WES also has been successfully used to identify susceptibility genes in cancer^45^,^46^. We acknowledge that there are potential limitations to our use of WES analysis. A main stumbling block is that WES targets only the protein-coding part (~1%) of the human genome, while leaving out microRNAs, lincRNAs, enhancers, repressors, certain promoters, and to a large extent, copy number variations. Moreover, due to technical difficulties, only about ~85% of exonic regions were covered. We applied WES only to two affected individuals, as mainly dictated by cost considerations, but note that such prohibitive costs have become more favorable recently. We required that candidate variants must be shared by both sequenced individuals, but acknowledge that this filter eliminated only a fraction of all false positives.

We applied several filtering steps to our WES and genotyping data, allowing us to narrow down the number of variants detected from several thousand generated by WES to a total number of 21 candidate variants (one in each of 21 genes) (Table 1). The first of these methods involved the application of linkage analysis on our genotyping data. This method is not commonly used anymore, because in individual families with four to six affected, closely related relatives, linkage “noise” leads to numerous peaks well below statistical significance, precluding the designation of a single locus as most likely to harbor a putative mutation. Therefore, we identified the areas on the linkage map that showed very low or negative linkage. We then ranked variants falling in these linkage-negative regions as low probability variants, so they were excluded from further analysis in this study. We note that systematic scrutiny of the efficacy of this method is still lacking.

Regarding our choices of downstream bioinformatics filters, we acknowledge that certain assumptions could result in false negatives. For example, our requirement that a variant would have to be pathogenic, as predicted by various criteria. could lead to the omission of certain candidates. On the other hand, determining the presence or absence of each candidate variant by Sanger sequencing in all relevant members of the family helped to validate the association of each candidate genotype with the PTC phenotype. Similarly, our study of haplotypes surrounding each candidate variant allowed us to eliminate 19 of our final 21 candidates (Table 1).

The likelihood that a given variant is causative could be assessed by case-control studies, where the variant’s frequency would be compared in further PTC cases (sporadic or familial or both) vs. in appropriate controls. The rationale for requiring that the studied variant should occur more often in familial PTC than in controls is based on expectations for an association between a particular genotype and the PTC phenotype. Moreover, if the variant were more prevalent in sporadic cases than in controls (and if the cases were certain to be sporadic), less-than-high penetrance would be suggested. For these reasons, we had to consider the numbers of cases and controls that needed to be typed. For example, if we assumed that only candidates with minor allele frequencies below 2% should be considered, then finding a variant in, say, 2 of 100 controls but in, say 7 of 100 cases would be a suggestive finding, but not a statistically significant one (p = 0.1697), in favor of such a candidate. Such findings then could be strengthened or weakened further by typing more cases and controls.

In evaluating the final 2 candidate variants in this study, we initially typed 187 cases and 189 controls. The *SRRM2* variant initially was found in two out of 187 cases and in zero out of 189 controls. When the numbers were expanded, the *SRRM2* variant was present in seven of 1,170 cases and in zero of 1,404 controls. This difference was statistically significant (p = 0.004), thereby strengthening the candidacy of this variant. In contrast, the *CHD9* variant was not detected either in 720 cases or in 727 controls. However, this negative result did not completely exclude this *CHD9* variant as a candidate in the family, as it could be causative but ultra-rare. Another comparable example was recently published, describing a SNP in an enhancer on chromosome 4 that accounts for PTC in a large US Midwestern family comprising 13 affected individuals. That SNP was not found in an Ohio cohort of 800 cases and 820 controls, nor in a Polish cohort of 1,876 cases and 1,650 controls^17^.

We noted an apparent contradiction in the penetrance of the candidate mutation in *SRRM2*. It displayed high penetrance in the affected family, but not in the families of the seven sporadic mutation carriers with PTC. Thus we concluded that this mutation displays autosomal dominant inheritance with incomplete or variable penetrance. Similar variable penetrance has been suggested before, for example in a *BRCA2* gene mutation^47^. This could occur by chance alone, but we cannot exclude the possibility that environmental factors or other genes interact with *SRRM2* in the PTC family (Fig. 1). This possibility should be the subject of further research.

We postulated that a variant in the splicing factor SRRM2, predicted to be probably damaging, might have an effect on AS. Measuring AS using RNA-Seq produced a large number of exons (n =1,642) which displayed significant differences in PSI levels between cases and controls. Interestingly, *SRRM2* is among a growing list of splicing components with disease associated variations. Other examples include mutations in factors that function early in spliceosome formation, such as mutations in the U2 snRNP auxiliary factor (U2AF), SRSF2 and the U2 snRNP SF3b1 protein, which have been linked to myelodysplastic syndromes that often progress to chemotherapy-resistant secondary acute myeloid leukemia^48^. Like the S346F mutation in PTC individuals, it has been proposed that these and other cancer-associated mutations have selective effects on AS, consistent with observations that reduced levels of core splicing components can impact the splicing of specific subsets of exons, typically harboring suboptimal splice sites^49^,^50^.

Finally, it should be noted that we were unable to obtain thyroid tissue from any individual with the S346F mutation in SRRM2. Thus all measurements of splicing were obtained from blood-derived RNA. We speculate that the mutated protein affects splicing of at least one of the genes specifically expressed in thyroid or involved in a thyroid-specific pathway, thereby leading to PTC formation.

## Materials and Methods

### Ethics statement

The experimental protocols were approved by the Institutional Review Board at the Ohio State University. All study participants gave written informed consent before participation. Methods were in accordance with the approved guidelines.

### Individuals and clinical samples

We defined “familial” as having at least two first- or second-degree relatives with PTC. Out of a total of 139 familial cases of PTC in our repository, we focused on one family displaying Mendelian-like inheritance in two generations (Fig. 1). This was one of the largest families not already subjected to in-depth genetic analyses. Family history information, pathology reports confirming the diagnosis of thyroid cancer or thyroid disease, and blood samples were collected from all consenting affected individuals and key unaffected individuals. Clinical information for the six affected family members and the seven sporadic cases with the *SRRM2* mutation is presented in Supplementary Table 2.

To investigate candidate predisposing genetic factors, we studied an additional 138 familial cases, 1,170 Ohio sporadic cases and 1,404 Ohio controls. The Ohio sporadic cases (n = 1,170) were individuals with PTC enrolled in the Ohio State University Wexner Medical Center’s (OSUWMC) endocrine neoplasia repository, a large repository of data and biological samples from individuals with thyroid neoplasia. Individuals were recruited from a multi-disciplinary thyroid tumor clinic at OSUWMC. All cases were histologically confirmed as PTC. Ohio control samples (n = 1,404), matched to cases by age, gender and race, were provided by the OSUWMC’s Human Genetics Sample Bank. The Columbus Area Controls Sample Bank provided additional control samples for use in human genetics research that included anonymized biological specimens and linked phenotypic data. Recruitment took place in OSUWMC primary care and internal medicine clinics. All study participants provided written informed consent, completed a questionnaire that included demographic, medical and family history information, and donated blood samples. Relevant clinicopathological data for cases were extracted from the electronic medical record. Demographic and clinical information for cases and controls is presented in Supplementary Table 4. Individuals from this pool of cases and controls were used in initial genotyping studies to determine the frequency of candidate mutations in cases vs. controls.

### DNA and RNA extraction

Genomic DNA was extracted from blood following standard phenol-chloroform extraction procedures. RNA was extracted from white blood cells using TRIzol reagen. RNA quality was checked using an Agilent 2100 Bioanalyzer (Supplementary Fig. 5).

### Genotyping

All individuals in the PTC family under study (Fig. 1) were genotyped using the HumanCytoSNP-12 v2.1 BeadChip array (lllumina lnc.) panel to assay 298,649 SNPs. GenomeStudio Genotyping Module Version 1.8.4 was used to analyze genotyping assay data. Low quality genotyping calls with low Gene Call scores (GC score < = 0.5) were treated as missing data. The average call rate with this cutoff was 92.3%. The Mendelian error rate was 0.1%. Errors were removed before further analysis.

### Whole-exome sequencing and analysis

Whole-exome sequencing (WES, 2 × 100-bp paired-end reads) was performed on exome-enriched DNA samples from two affected individuals in the PTC family under study. We used the HiSeq 2000 platform in the Biomedical Genomics Core of Nationwide Children’s Hospital, Columbus, OH, as part of the Illumina Genome Network (IGN; http://www.illumina.com). Exomes were enriched using Agilent SureSelect Human All Exon kit.

Raw Illumina sequence reads were aligned against the reference human genome (UCSC hg19) using BWA^51^. Duplicate reads were removed, and reads with an alignment score <30 also were filtered out. The mean depth of exome sequencing coverage was 50x (range from 41x to 54x coverage). Greater than 75% of target regions were sequenced at 20x or greater depth of coverage. Genomic variants were called using Samtools/BCFtools^52^. Only those variants which were present in both patients and which occurred at <2% population allele frequency in the 1000 Genomes database were considered further.

Predicted functional consequences of the variants were annotated using Ensembl Variant Effect Predictor^53^. We focused on variants with pathogenic consequences (missense, stop-gain, splice site, and frameshift mutations). We also eliminated genes found not to be expressed in thyroid tissue using our previous gene expression microarray data^26^ and data at http://cgap.nci.nih.gov/SAGE/AnatomicViewer. Variants were further filtered using criteria described in Results. dbGaP submission of WES data is pending (working on amending the IRB protocol that does not approve sharing the data).

### DNA resequencing and screening for candidate mutations

Blood genomic DNA from seven available individuals of the family (Fig. 1) was used for Sanger resequencing. PCR primer sequences are shown in Supplementary Table 2. PCR assays were performed according to a standard protocol using AmpliTaq Gold DNA polymerase (Life Technologies) as follows: 10 min at 94 °C; followed by 32 cycles of 15 s at 94 °C, 15 s at 58 °C, and 1 min at 72 °C; followed by a final extension of 5 min at 72 °C. The PCR amplicons were sequenced using an ABI3730 DNA sequencer (Genomics Shared Resource, OSU).

SNaPshot assays (Life Technologies) were performed as described^18^. To amplify genomic regions around the 2 candidate variants (*CHD9, SRRM2*), PCR was performed followed by a single nucleotide extension reaction.

Screening for the SNP rs149019598 in Ohio PTC patients and controls was carried out by deCODE Genetics in Reykjavik, Iceland, applying the Centaurus (Nanogen) single-track assay^54^. DNA that had been whole-genome amplified using standard protocols based on the Phi29 polymerase (Thermo Fisher Scientific Inc.) was used. To confirm the T allele at rs149019598 as identified from the single-track assay, we used Sanger sequencing to assay unamplified DNA extracted from whole blood.

### RNA-Seq sample preparation

RNA-Seq experiments were performed as described previously^55^. Briefly, 10 ug extracted total RNA was DNase-treated (Turbo DNase, Ambion) and polyadenylated RNA was enriched twice from total RNA using polyATract mRNA isolation system IV (Promega). RNA was reverse transcribed and converted into double-stranded cDNA (SuperScript cDNA synthesis kit, Invitrogen), sheared by sonication followed by end-repairment, A-tailing, paired and end adapter (Illumina) ligation. Prior to PCR amplification, cDNA was UNG treated to maintain strand-specificity. cDNA was amplified by 15 cycles of PCR and size selected (200-300 bp). The total number of sequencing reads per sample was 160-170 million, and the average read length was ~100 nucleotides. dbGaP submission of RNA-Seq data is pending (working on amending the IRB protocol that does not approve sharing the data).

### RNA-Seq analysis

To identify exons undergoing alternative splicing, we calculated “percent spliced in” (PSI) values from RNA-Seq data, essentially as previously described^36^. Briefly, we considered every internal exon in each annotated transcript a potential “cassette” exon. Each alternative splicing event involving such a cassette was defined by three exons: C1, A and C2 (Supplementary Fig.3) where A is the alternative exon, C1 is the 5´ constitutive exon and C2 is the 3´ constitutive exon. For each event we define two constitutive junctions, C1A (connecting exons C1 and A) and AC2 (connecting exons A and C2), and one alternative (or “skipped”) junction, C1C2 (connecting exons C1 and C2). The percent inclusion, or “percent spliced-in” (PSI) value, for each internal exon was defined as: PSI = 100 × average(#C1A,#AC2) / (#C1C2 + average(#C1A,#AC2)), where #C1A, #AC2 and #C1C2 are the normalized read counts for the associated junctions.

PSI values were determined for three cases and three controls. AS predictions were made in those cases where at least 50 reads mapped to the C1C2 junction or (> = 50 reads mapped to max (C1A, AC2) junctions and > = 35 reads mapped to min (C1A, AC2) junctions) in all six samples, where AS exons were as defined in the Results.

To validate PSI-based predictions of differential AS in cases vs. controls, we next selected five cases, all carrying the heterozygous c.1037C >T mutation, and nine wild-type control samples. We required the difference between the average PSI values for the two groups to be > = 20%. Confirmatory RT-PCR assays (primer sequences available in Supplementary Table 2) were performed. PCR products were visualized upon electrophoresis in 1.5% agarose gels by staining with ethidium bromide (Fig. 4a). The two AS bands were quantified using AlphaView SA software (Proteinsimple, Santa Clara, CA). In these confirmatory experiments, the PSI was calculated as the ratio of the AS isoform including the alternative exon A, divided by the sum of the isoforms “including” and “excluding” the alternative exon.

### Statistical analysis

Genome-wide non-parametric linkage analysis was performed with MERLIN 1.1.2 software^56^. Haplotypes were constructed using MERLIN, and were visualized using Haplopainter^57^.

In comparing PSI values between PTC patients that are heterozygous for the mutation and controls, we applied the t-test statistic after arcsin(sqrt) transformation of proportion data. Cluster analysis of samples using significantly different events with P < 0.05 was performed using the Pearson correlation similarity metric and using an average linkage method.

## Additional Information

**How to cite this article**: Tomsic, J. *et al*. A germline mutation in *SRRM2*, a splicing factor gene, is implicated in papillary thyroid carcinoma predisposition. *Sci. Rep.* **5**, 10566; doi: 10.1038/srep10566 (2015).

## Supplementary Material

Supplementary Information

## Figures and Tables

**Table 1:** Candidate predisposing variants identified by WES and evaluated by haplotype sharing.

Gene	Ensembl Gene	Ensembl Transcript	Chromosomal position	Nucleotide (cDNA)	Amino acid (protein)	MAF (%) (ESP/1K)*	dbSNP	Haplotype sharing
MIER1	ENSG00000198160	ENST00000355356	1:67450431	c.1387A>G	p.R463G	2.6/2.09	rs17129563	NO
HECW2	ENSG00000138411	ENST00000260983	2:197183332	c.2282G>A	p.G761E	0.27/NA	rs61752162	NO
ADH1B	ENSG00000196616	ENST00000305046	4:100229021	c.1104T>G	p.S368R	0.1/0.1	rs74990410	NO
FAT4	ENSG00000196159	ENST00000394329	4:126412106	c.14129C>G	p.S4710C	2.06/1.79	rs147662558	NO
BTN3A3	ENSG00000111801	ENST00000244519	6:26452211	c.1327C>T	p.R443W	0.92/0.99	rs148819206	NO
HLA-DPA1	ENSG00000231389	ENST00000419277	6:33037060	c.364G>A	p.V122M	0.85/0.80	rs115722167	NO
ENPP1	ENSG00000197594	ENST00000360971	6:132211530	c.2657G>C	p.R886T	0.59/0.40	rs8192683	NO
MTUS1	ENSG00000129422	ENST00000262102	8:17611593	c.1724TA>G	p.H575R	0.85/1.09	rs209569	NO
PSD3	ENSG00000156011	ENST00000286485	8:18662355	c.86C>T	p.T29I	0.13/0.10	rs142032665	NO
SUPT20H	ENSG00000102710	ENST00000360252	13:37596401	c.1645C>T	p.H549Y	NA/NA	NA	NO
COG6	ENSG00000133103	ENST00000416691	13:40253690	c.649C>A	p.L217M	NA/NA	NA	NO
NIN	ENSG00000100503	ENST00000382041	14:51206145	c.5509T>A	p.S1837T	3.37/2.98	rs12717411	NO
SRRM2	ENSG00000167978	ENST00000301740	16:2811566	c.1037C>T	p.S346F	0.09/0	rs149019598	**YES**
ABCC6	ENSG00000091262	ENST00000205557	16:16295863	c.1171A>G	p.R391G	0.92/0.99	rs72653762	NO
DNAH3	ENSG00000158486	ENST00000261383	16:21098323	c.2724C>G	p.R908S	0.77/0.7	rs117470111	NO
SCNN1G	ENSG00000166828	ENST00000300061	16:23203830	c.776C>A	p.T259N	0.09/NA	rs72646501	NO
CHD9	ENSG00000177200	ENST00000566029	16:53190063	c.62G>A	p.G21D	NA/NA	NA	**YES**
SYNRG	ENSG00000275066	ENST00000612223	17:35937577	c.724A>G	p.M242V	0.02/0	rs143491502	NO
ZNF507	ENSG00000168813	ENST00000311921	19:32845600	c.1864G>A	p.D622N	1.16/1.39	rs117938843	NO
PTGIS	ENSG00000124212	ENST00000244043	20:48156146	c.634C>T	p.R212W	0/0	rs201920776	NO
IDS	ENSG00000010404	ENST00000340855	X:148578002	c.754G>A	p.D252N	0.36/0.7	rs146458524	NO

Gene, transcript and chromosomal positions taken from Ensembl build 37 (http://www.ensembl.org)

ref = reference allele in genomic DNA; alt = alternate allele; MAF = minor allele frequency;

CDS = coding DNA sequence; NA = not available; haplotype sharing = estimation of haplotypes using Merlin.

Data for variants surrounding the mutation were available for 7 individuals, while information about mutation was available only for 2 affected individuals.

^*^MAF as reported by ESP (NHLBI Exome Sequencing Project) in European American population and 1K (1000 Genomes phase 3) genotyping data in European population (NA when data not available across populations).

**Table 2:** Validation of PSI in genes determined by RNA-Seq, using RT-PCR.

	Average PSI, RNA-Seq*		Average PSI, RT-PCR*	
Events	Cases (n = 3)	Controls (n = 3)	Cases (n = 5)	Controls (n = 9)
CAMKK2:NM_172226 (exon 14)	48.1	23.9	41.8	28
CTNNA1:NM_001903 (exon 3)	91.7	70.6	79.8	72.7
CDC16:NM_003903 (exon 17)	83	61.2	85	79.4
FBXW4:NM_022039 (exon 7)	85.9	64.7	75.2	67.8
HBP1:NM_012257 (exon 7)	65.3	38.4	81.4	53.4
PIM2:NM_006875 (exon 5)	72.3	49.6	71	46.7
SPPL3:NM_139015 (exon 10)	78.5	56.3	80.4	69

Seven genes which displayed differences of >20% in RNA-Seq between cases and controls, were evaluated using PSI determined by RT-PCR for cases and controls.

^*^These PSI values (calculated as described in Materials and Methods) were used in the scatter plot (Supplementary Figure 3).
