# Supplementary material for: A germline mutation in SRRM2, a splicing factor gene, is implicated in papillary thyroid carcinoma predisposition
Source: Sci Rep. 2015 Jul 2;5:10566. doi: 10.1038/srep10566 (PMC4488885; doi:10.1038/srep10566)
Supplement: Supplementary Information [file srep10566-s1.pdf]

A germline mutation in *SRRM2*, a splicing factor gene, is implicated in papillary thyroid carcinoma predisposition

Jerneja Tomsic[1], Huiling He[1], Keiko Akagi[1], Sandya Liyanarachchi[1], Qun Pan[2], Blake Bertani[1], Rebecca Nagy[3], David E. Symer[1,3,4], Benjamin J. Blencowe[2,5] & Albert de la Chapelle[1]

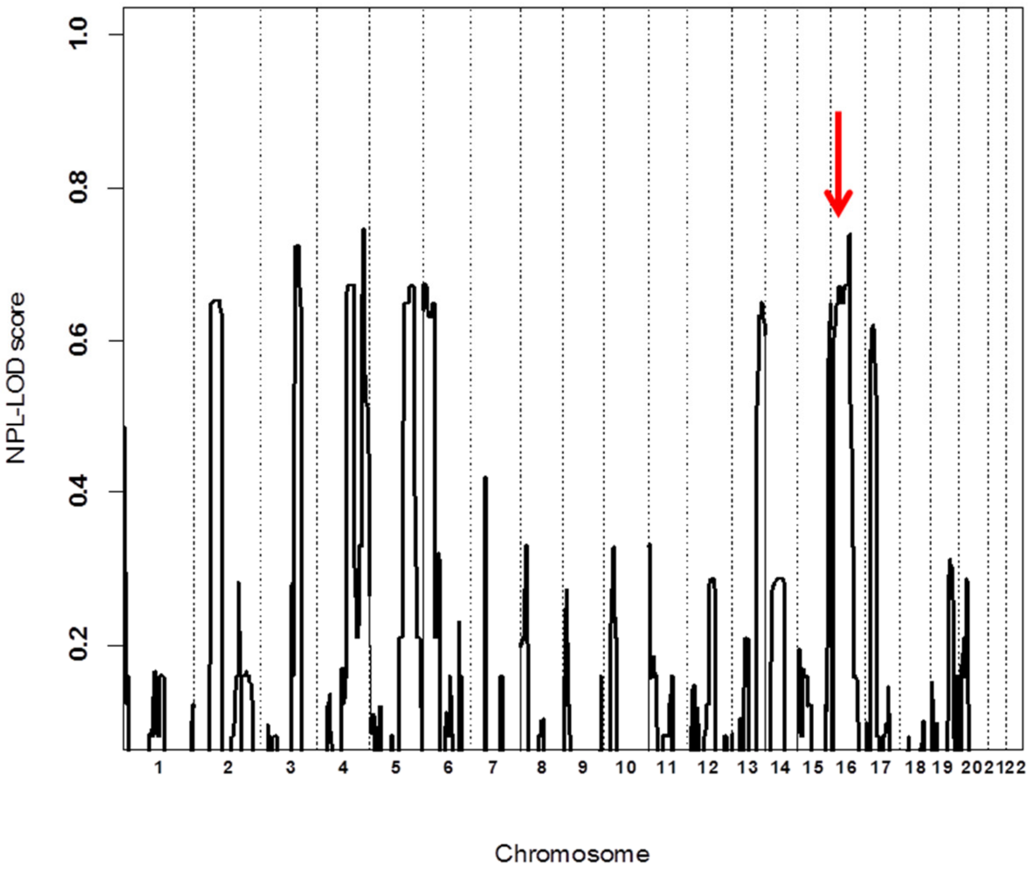

Supplementary Figure 1. Linkage analysis of Family 7.
Red arrow, position of *SRRM2* gene in chromosome16.

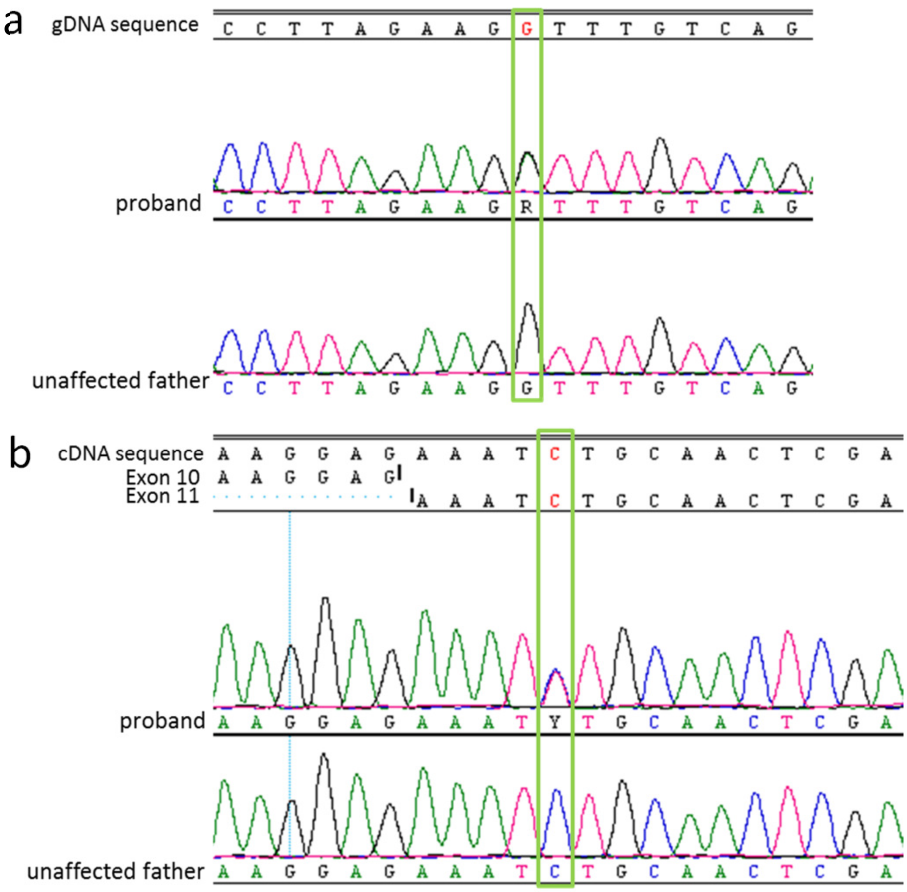

Supplementary Figure 2. Sanger sequencing of the two variants (*CHD9* and *SRRM2*).
(**a**) Chromatogram of region around *CHD9* variant in the proband and unaffected father using gDNA from blood. SRRM2 variant was confirmed in the same individuals and it confirmed the WES findings. (**b**) Chromatogram of the region around the *SRRM2* variant (c.1037C>T). We reverse transcribed blood RNA and Sanger sequenced the region spanning exon9-exon11 in order to confirm the presence of the variant. The presence of the *SRRM2* variant was confirmed in the blood RNA from the proband. Although this mutation so close to the 5'-end of an exon was not predicted to affect the splice site, this sequencing of RNA confirmed the presence of both alleles leading to the expression of the two variants of SRRM2 protein.

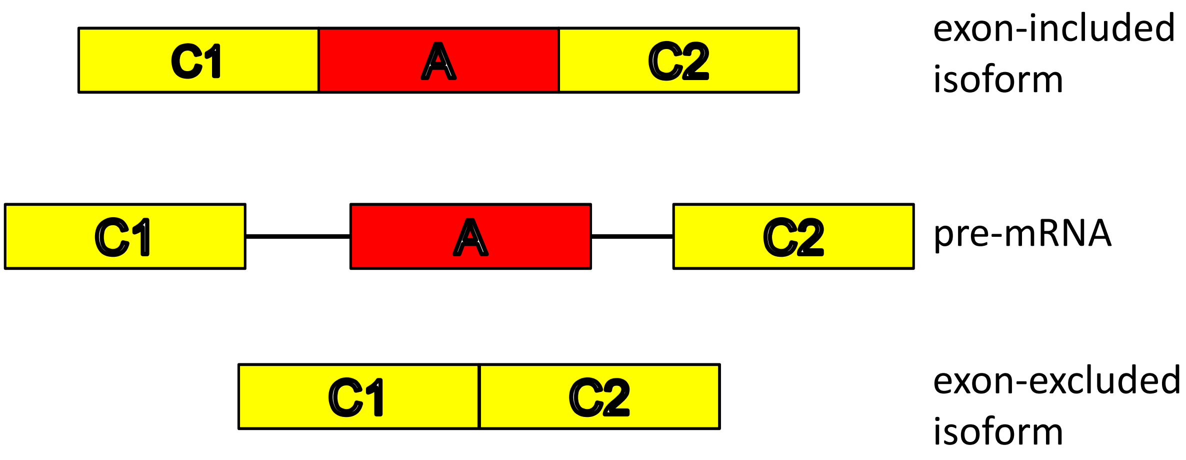

Supplementary Figure 3. Schematic representation of alternative splicing. Using RNA-Seq we analyzed the presence of junctions C1A, AC2 and C1C2 in our samples. PSI values were calculated for all the internal exons (A) based on the junctions present as described in Barbosa-Morais *et al*., Science (2012), the manuscript cited in Materials and Methods.

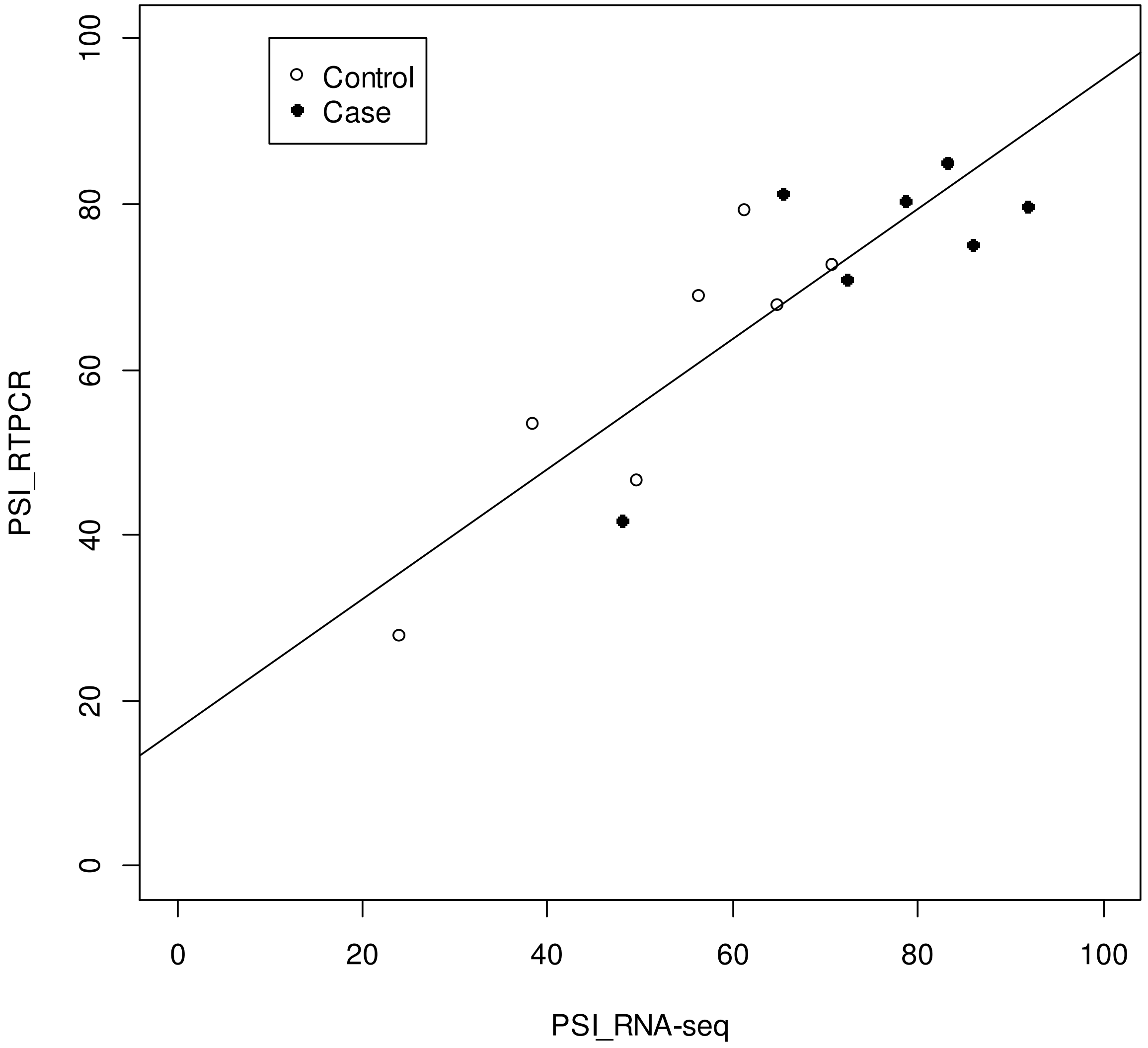

Supplementary Figure 4. Correlation between RT-PCR –estimated and RNA-Seq –estimated “percent spliced in” (PSI) values.
Scatter plot showing correlation between the 2 methods in each of the seven genes tested. PSI for the same seven genes was measured in cases and controls. Each dot represents an average of 5 values for cases and an average of 7 values for controls. Due to the small numbers (7 genes studied in cases and same 7 genes studied in controls) we decided to analyze all of these 14 values together. Pearson correlation: all samples: cor=0.865, p-value=6.373e-05; Spearman rank correlation: all samples cor=0.798, p-value=0.001

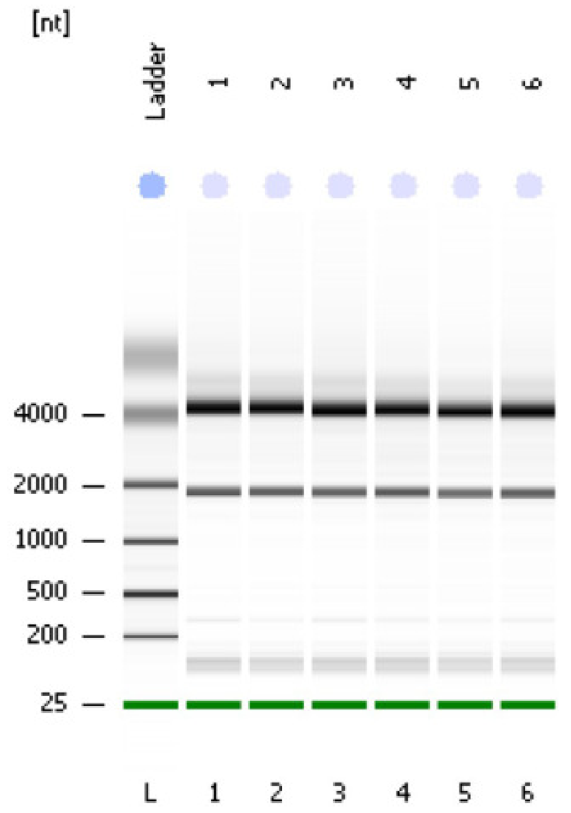

Supplementary Figure 5. Blood RNA quality control.
After blood RNA was extracted using the standard Trizol method the quality of RNA was assessed via Agilent's 2100 Bioanalyzer. RIN values were 9.8 or above. RNA samples from cases (1-3) and controls (4-6) were sent for RNA-Seq analysis to Donnelly Sequencing Centre at the University of Toronto. Samples were treated in the same way as described extensively by Barbosa-Morais *et al.*, Science (2012), the manuscript cited in Materials and Methods.

Supplementary Table 1. Clinical features of SRRM2 mutation positive cases

Case #	Gender	Race	Histologic sub-type	Age	T stage	N stage	M stage	Familial/Sporadic
1	M	Caucasian	PTC, classic	45	PT2	N1	M0	S
2	F	Caucasian	microPTC	46	PT1	N0	M0	S
3	F	Caucasian	PTC,fv	56	PT1	N0	M0	S
4	F	Caucasian	PTC,fv	25	PT2	N0	M0	S
5	F	Caucasian	PTC,fv	51	PT2	N0	M0	S
6	M	Caucasian	PTC,fv	68	PT3	N0	M0	S
7	F	Caucasian	microPTC	69	PT1	N0	M0	S
8*	F	Caucasian	microPTC	25	PT1	N0	M0	F
9*	F	Caucasian	microPTC	51	PT1	N0	M0	F
10*	F	Caucasian	PTC, classic	23	PT1	N1	M0	F
11*	F	Caucasian	PTC, classic	17	PT1	N0	M0	F
12*	F	Caucasian	microPTC	58	PT1	N0	M0	F
13*	F	Caucasian	PTC,fv	57	PT2	N0	M0	F

PTC = papillay thyroid cancer; microPTC = PTC less than 1.0 cm in greatest dimension; PTC, fv = PTC follicular variant; Age = age at diagnosis of PTC * Cases 8-13 are members of the family.

Supplementary Table 2. Primers used in PCR reactions.

PCR primers to test for the variants (Sanger sequencing):	
CHD9_fw	TTGACACTTCATGACATGTC
CHD9_rv	CTGAGAGTGTGGAGATAACAC
SRRM2_fw	AAGTGATCGCTTGTGGTCAG
SRRM2_rv	AAGTTTCTCGGGAGACTTAG
PCR primers to confirm alternative splicing data via end-point RT-PCR:	
CAMKK2_F	GCCCGACATAGCTGAGGACT
CAMKK2_R	AGCAAGTTTCCAGGCGCTGAC
CDC16_F	ATGCTGAGGCCTTGGATTACCAC
CDC16_R	TGAGGTTTCCAATGGCGTAAGCC
CTNNA1_F	ATGCAGGCAACATAAACTTCAAGTG
CTNNA1_R	TCAGCTGAACAAGTAATTTGTAGACATC
FBXW4_F	GACAGGGACGGCTTGTTGCG
FBXW4_R	TGCAGGCAGGCCCTTTGACG
HBP1_F	ACACGACTGTGCTTTCATAAGGG
HBP1_R	TCCAGGAGGTAGACATACATCGC
PIM2_F	CCATCGTGACATCAAGGATG
PIM2_R	TCCTCAATCCCTTACCTTAG
SPPL3_F	TCCACTGGCAGCCACTTCTC
SPPL3_R	AGAAGGTAACAGTCTGGTGCAGA

Supplementary Table 3. Testing for "Percent spliced in" (PSI) in 7 genes displaying >20% difference in RNA-Seq - estimated PSI between cases and controls.

	Average PSI RNA-Seq*		Average PSI RT-PCR*	
Events	Cases (n=3)	Controls (n=3)	Cases (n=5)	Controls (n=9)
CAMKK2:NM_172226:14	48.1	23.9	41.8	28.0
CTNNA1:NM_001903:3	91.7	70.6	79.8	72.7
CDC16:NM_003903:17	83.0	61.2	85.0	79.4
FBXW4:NM_022039:7	85.9	64.7	75.2	67.8
HBP1:NM_012257:7	65.3	38.4	81.4	53.4
PIM2:NM_006875:5	72.3	49.6	71.0	46.7
SPPL3:NM_139015:10	78.5	56.3	80.4	69.0

* These PSI values were used in the scatter plot (Supplementary Figure 5).

Supplementary Table 4. Clinical and demographic information on cases and controls.

	Cases N (%)	Controls N (%)
**Gender**		
**Female**	898 (77)	1069 (76)
**Male**	272 (23)	335 (24)
**Race**		
**Caucasian**	1097 (93.8)	1317 (93.8)
**African American**	42 (3.6)	54 (3.8)
**Asian**	31 (2.6)	33 (2.4)
**Mean age^**	41.4 yrs (range 7-88 yrs)	43.8 yrs (range 18-87 yrs)
**Histologic sub-type**		
**PTC, classic type**	680	
**PTC, follicular variant**	240	
**microPTC**	207	
**PTC, other**	43	
**Total**	1170 (100)	1404(100)

^ Mean age of cases is age at diagnosis of thyroid cancer; mean age for controls is age at time of study enrollment and blood draw.

PTC = papillary thyroid carcinoma; microPTC = PTC less than 1.0 cm in greatest dimension
